# The genome sequence of the common buff snailkiller, *Tetanocera ferruginea *(Fallén, 1820)

**DOI:** 10.12688/wellcomeopenres.20366.1

**Published:** 2023-11-20

**Authors:** Liam M. Crowley, Steven Falk, Christopher D. Williams, University of Oxford and Wytham Woods Genome Acquisition Lab, Darwin Tree of Life Barcoding collective, Wellcome Sanger Institute Tree of Life programme, Wellcome Sanger Institute Scientific Operations: DNA Pipelines collective, Tree of Life Core Informatics collective, Darwin Tree of Life Consortium

**Affiliations:** 1 Department of Biology, University of Oxford, Oxford, England, UK; 2 Independent researcher, Kenilworth, England, UK; 3 Biological and Environmental Sciences, Liverpool John Moores University, Liverpool, England, UK

**Keywords:** Tetanocera ferruginea, common buff snailkiller, genome sequence, chromosomal, Diptera

## Abstract

We present a genome assembly from an individual male *Tetanocera ferruginea* (the common buff snailkiller; Arthropoda; Insecta; Diptera; Sciomyzidae). The genome sequence is 790.4 megabases in span. Most of the assembly is scaffolded into 7 chromosomal pseudomolecules, including the X and Y sex chromosomes. The mitochondrial genome has also been assembled and is 17.07 kilobases in length.

## Species taxonomy

Eukaryota; Metazoa; Eumetazoa; Bilateria; Protostomia; Ecdysozoa; Panarthropoda; Arthropoda; Mandibulata; Pancrustacea; Hexapoda; Insecta; Dicondylia; Pterygota; Neoptera; Endopterygota; Diptera; Brachycera; Muscomorpha; Eremoneura; Cyclorrhapha; Schizophora; Acalyptratae; Sciomyzoidea; Sciomyzidae; *Tetanocera*; *Tetanocera ferruginea* (Fallén, 1820) (NCBI:txid320963).

## Background

Since the seminal discovery of obligate malacophagy (mollusc-feeding) in the dipteran family Sciomyzidae by ( Berg, 1953), there has been a steady increase in our knowledge of the family ( Berg & Knutson, 1978; Murphy *et al.*, 2012), so that today, lifecycles are known in 203 species out of a total described number of species of 539 (38% of the family) making Sciomyzidae among the most biologically well-known dipterous families in the world ( Knutson & Vala, 2011). *Tetanocera ferruginea* Fallén, 1820 is a medium to large fly, rather drab in colour being light brown with no obvious wing markings other than those common to all *Tetanocera* species. It is not easily distinguished from *Tetanocera fuscinervis* (Zetterstedt, 1838) or other *Tetanocera* species. It has shorter legs than *Tetanocera hyalipennis* von Roser, 1840, is smaller than *Tetanocera robusta* Loew, 1847, but reliable identification requires dissection of male genitalia. Good figures of male postabdomen morphology are given in Rozkošný (1984), Rozkošný (1987) and Vala (1989). Naturhistoriska Riksmuseet, Stockholm, Sweden held the holotype, but it is presumed lost ( Vala *et al.*, 2012).

*Tetanocera ferruginea* has a Holarctic distribution ( Vala *et al.*, 2012) with distribution maps provided in Foote (1999), Sueyoshi (2001), Williams *et al*. (2007) and McDonnell *et al.* (2010). It is not considered scarce or threatened in the UK ( Falk, 1991). The biology of *T. ferruginea* is discussed in some detail in Foote (1961), Rozkošný (1965), and Vala (1989). It was placed in Phenological Group 1 by Berg *et al*. (1982). This Group is defined as follows: “Multivoltine species overwintering in the puparium as diapausing or quiescent prepupae, pupae, or pharate adults. The puparial stage is found throughout the year” ( Vala *et al.*, 2012). Knutson and Vala (2011) placed *T. ferruginea* in Behavioural Group 11. This Group is defined as follows: “Predators of non-operculate snails at or just below the water surface, just above the surface on emergent vegetation, and occasionally on snails exposed on moist, ‘shoreline’ surfaces” ( Vala *et al.*, 2012). It has been shown to have very limited movements within habitats despite large populations ( Williams *et al.*, 2010)

This genome sequence will be extremely useful for applying Rad-Seq analysis to the population genetics of *T. ferruginea*, as suggested by Williams (2023). Previous population genetics studies of Sciomyzidae are limited to a 1990 isozyme study of the *Sepedon fuscipennis* group ( Manguin, 1990). More recent population genetics work has attempted to study *Tetanocera ferruginea* but failed to produce sufficient specimens. It is not clear yet whether this is due to a general global decline in the species or a temporary bottleneck.

## Genome sequence report

**Figure 1.**
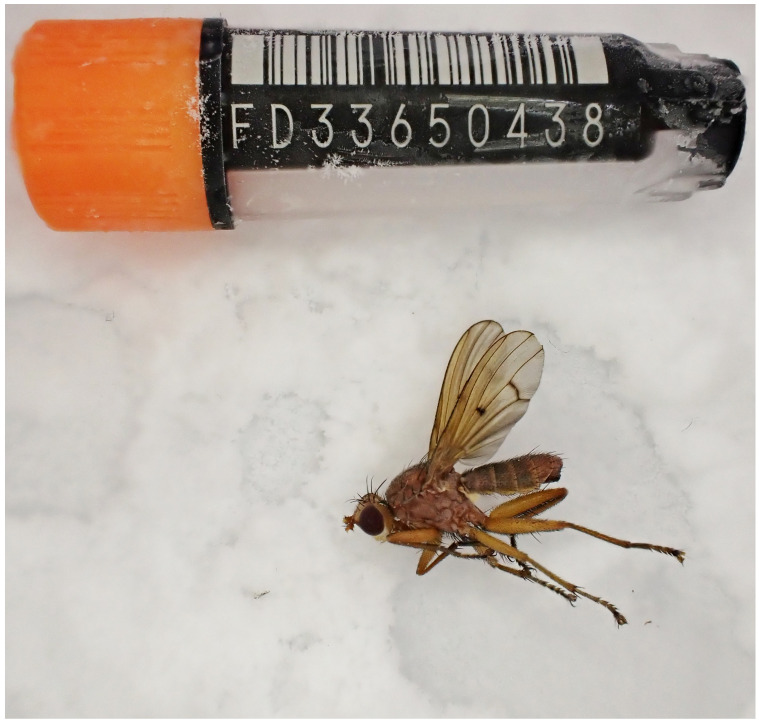
Photograph of the *Tetanocera ferruginea* (idTetFerr1) specimen used for genome sequencing.

The genome was sequenced from one male *Tetanocera ferruginea* ( Figure 1) collected from Wytham Woods, Oxfordshire (biological vice-county Berkshire), UK (51.76, –1.34). A total of 30-fold coverage in Pacific Biosciences single-molecule HiFi long reads was generated. Primary assembly contigs were scaffolded with chromosome conformation Hi-C data. Manual assembly curation corrected 87 missing joins or mis-joins and removed 2 haplotypic duplications, reducing the scaffold number by 31.4%, and increasing the scaffold N50 by 19.24%.

**Figure 2.**
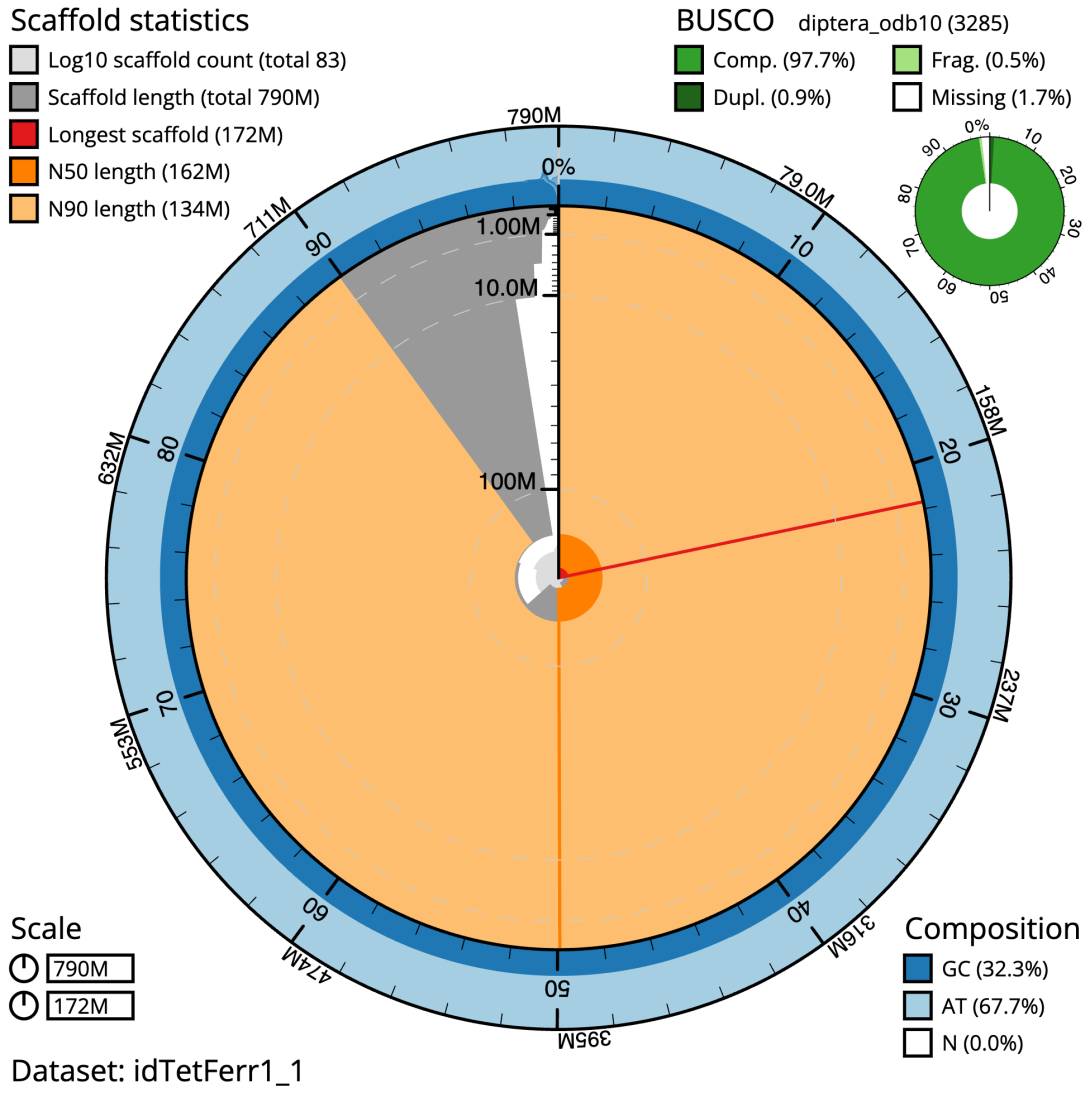
Genome assembly of *Tetanocera ferruginea*, idTetFerr1.1: metrics. The BlobToolKit Snailplot shows N50 metrics and BUSCO gene completeness. The main plot is divided into 1,000 size-ordered bins around the circumference with each bin representing 0.1% of the 790,383,743 bp assembly. The distribution of scaffold lengths is shown in dark grey with the plot radius scaled to the longest scaffold present in the assembly (172,084,289 bp, shown in red). Orange and pale-orange arcs show the N50 and N90 scaffold lengths (161,584,466 and 133,911,736 bp), respectively. The pale grey spiral shows the cumulative scaffold count on a log scale with white scale lines showing successive orders of magnitude. The blue and pale-blue area around the outside of the plot shows the distribution of GC, AT and N percentages in the same bins as the inner plot. A summary of complete, fragmented, duplicated and missing BUSCO genes in the diptera_odb10 set is shown in the top right. An interactive version of this figure is available at https://blobtoolkit.genomehubs.org/view/Tetanocera%20ferruginea/dataset/idTetFerr1_1/snail.

**Figure 3.**
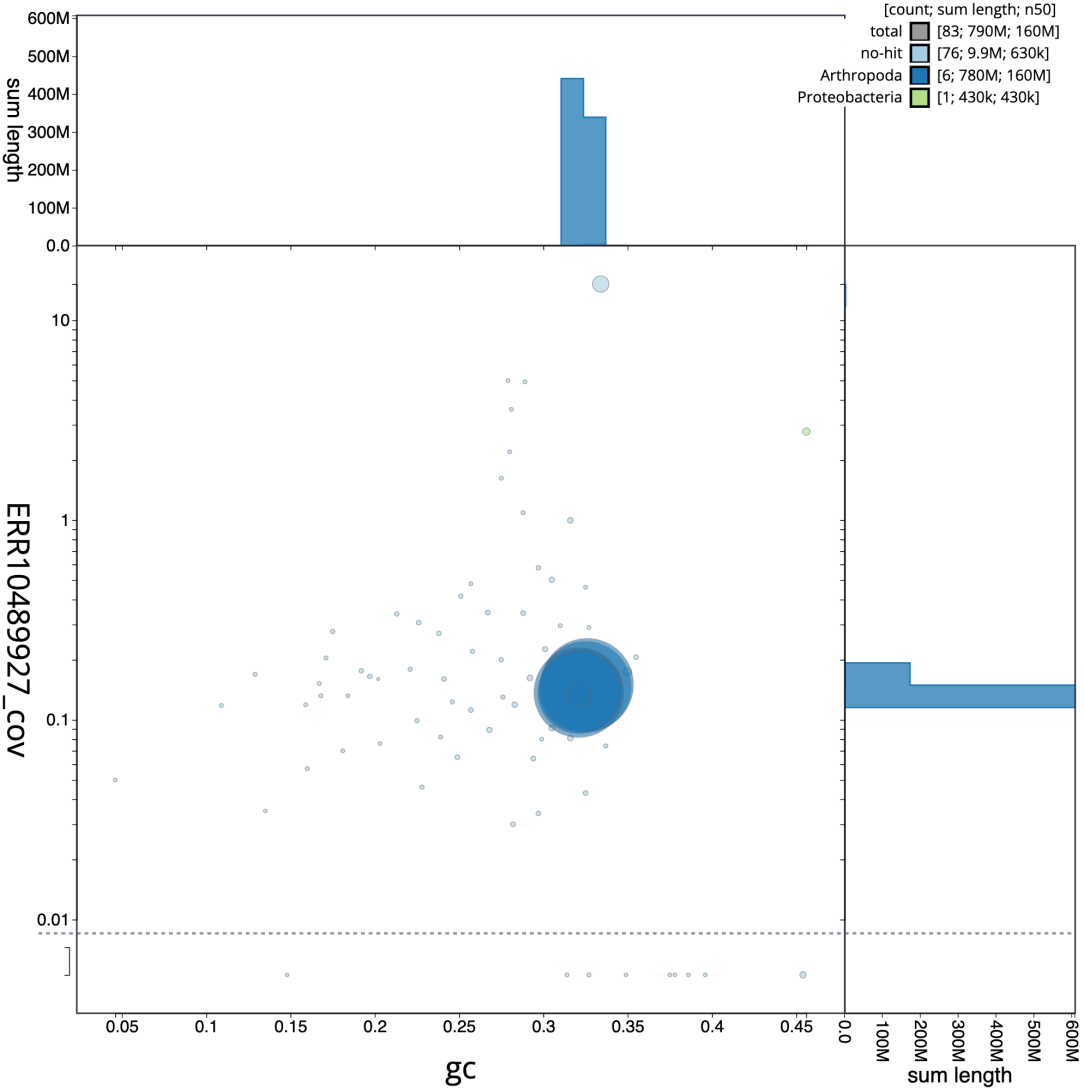
Genome assembly of *Tetanocera ferruginea*, idTetFerr1.1: BlobToolKit GC-coverage plot. Scaffolds are coloured by phylum. Circles are sized in proportion to scaffold length. Histograms show the distribution of scaffold length sum along each axis. An interactive version of this figure is available at https://blobtoolkit.genomehubs.org/view/Tetanocera%20ferruginea/dataset/idTetFerr1_1/blob.

**Figure 4.**
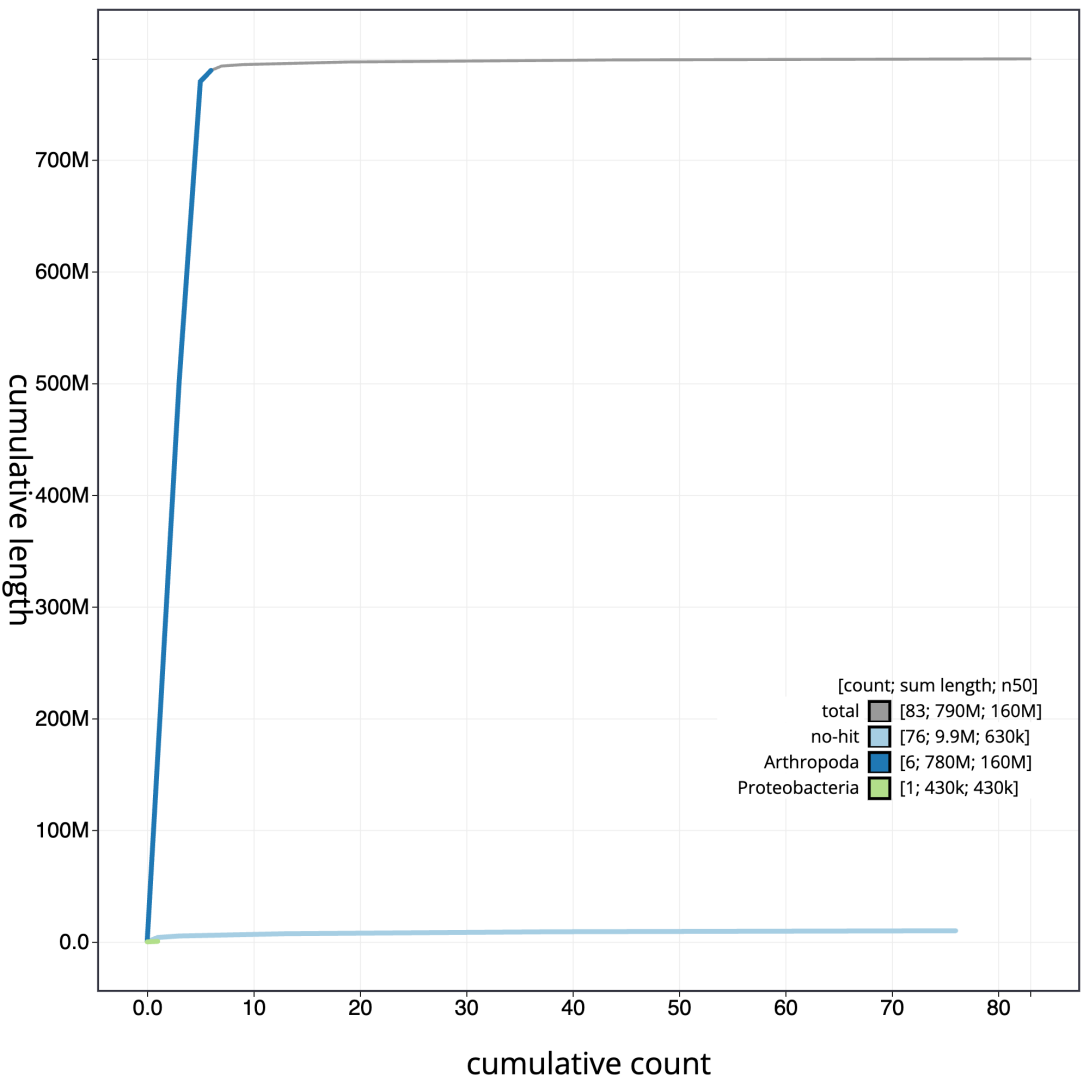
Genome assembly of *Tetanocera ferruginea*, idTetFerr1.1: BlobToolKit cumulative sequence plot. The grey line shows cumulative length for all scaffolds. Coloured lines show cumulative lengths of scaffolds assigned to each phylum using the buscogenes taxrule. An interactive version of this figure is available at https://blobtoolkit.genomehubs.org/view/Tetanocera%20ferruginea/dataset/idTetFerr1_1/cumulative.

**Figure 5.**
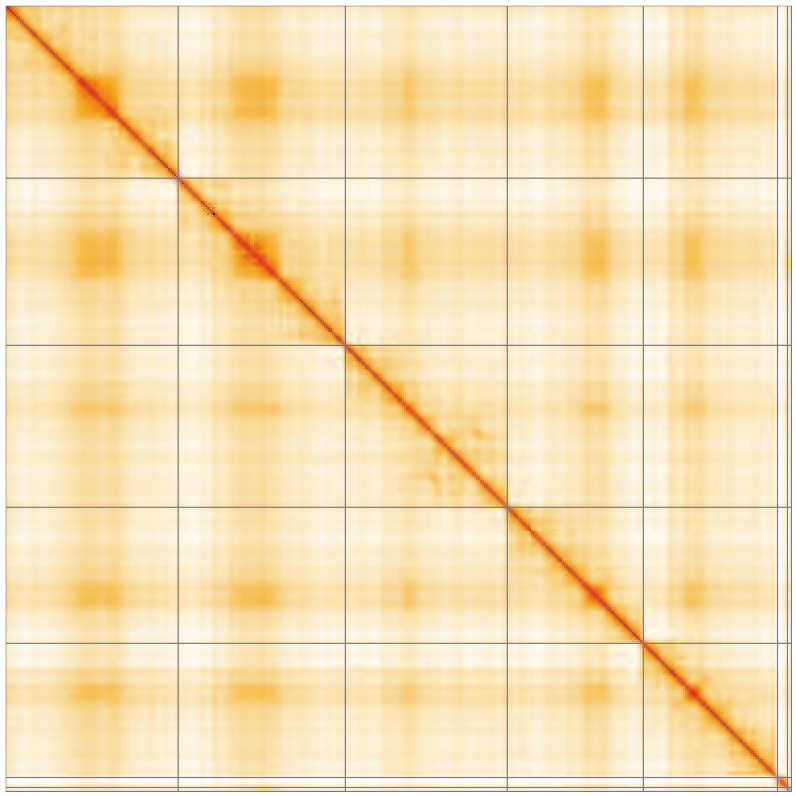
Genome assembly of *Tetanocera ferruginea*, idTetFerr1.1: Hi-C contact map of the idTetFerr1.1 assembly, visualised using HiGlass. Chromosomes are shown in order of size from left to right and top to bottom. An interactive version of this figure may be viewed at https://genome-note-higlass.tol.sanger.ac.uk/l/?d=BnOn57OCRVWnD40LgRLOKg.

The final assembly has a total length of 790.4 Mb in 82 sequence scaffolds with a scaffold N50 of 161.6 Mb ( Table 1). The snailplot in Figure 2 provides a summary of the assembly statistics, while the distribution of assembly scaffolds on GC proportion and coverage is shown in Figure 3. The cumulative assembly plot in Figure 4 shows curves for subsets of scaffolds assigned to different phyla. Most (99.18%) of the assembly sequence was assigned to 7 chromosomal-level scaffolds, representing 5 autosomes and the X and Y sex chromosomes. The sex chromosomes were determined by coverage statistics and synteny to *Pherbina coryleti* (GCA_943735915.1) ( Sivell *et al.*, 2023) and *Coremacera marginata* (GCA_914767935.1) ( Sivell *et al.*, 2021). Chromosome-scale scaffolds confirmed by the Hi-C data are named in order of size ( Figure 5; Table 2). While not fully phased, the assembly deposited is of one haplotype. Contigs corresponding to the second haplotype have also been deposited. The mitochondrial genome was also assembled and can be found as a contig within the multifasta file of the genome submission.

**Table 1.** Genome data for *Tetanocera ferruginea*, idTetFerr1.1.

Project accession data		
Assembly identifier	idTetFerr1.1	
Assembly release date	2023-07-06	
Species	*Tetanocera ferruginea*	
Specimen	idTetFerr1	
NCBI taxonomy ID	320963	
BioProject	PRJEB61619	
BioSample ID	SAMEA112232884	
Isolate information	idTetFerr1, whole organism (DNA sequencing and Hi-C data)	
Assembly metrics *		*Benchmark*
Consensus quality (QV)	57.2	*≥ 50*
*k*-mer completeness	99.99%	*≥ 95%*
BUSCO **	C:97.7%[S:96.8%,D:0.9%], F:0.5%,M:1.7%,n:3,285	*C ≥ 95%*
Percentage of assembly mapped to chromosomes	99.18%	*≥ 95%*
Sex chromosomes	X and Y chromosomes	*localised homologous pairs*
Organelles	Mitochondrial genome assembled	*complete single alleles*
Raw data accessions		
PacificBiosciences SEQUEL II	ERR11279092	
Hi-C Illumina	ERR11271535	
Genome assembly		
Assembly accession	GCA_958299015.1	
*Accession of alternate haplotype*	GCA_958298955.1	
Span (Mb)	790.4	
Number of contigs	519	
Contig N50 length (Mb)	3.9	
Number of scaffolds	82	
Scaffold N50 length (Mb)	161.6	
Longest scaffold (Mb)	172.1	

* Assembly metric benchmarks are adapted from column VGP-2020 of “Table 1: Proposed standards and metrics for defining genome assembly quality” from ( Rhie *et al.*, 2021). ** BUSCO scores based on the diptera_odb10 BUSCO set using v5.3.2. C = complete [S = single copy, D = duplicated], F = fragmented, M = missing, n = number of orthologues in comparison. A full set of BUSCO scores is available at https://blobtoolkit.genomehubs.org/view/Tetanocera%20ferruginea/dataset/idTetFerr1_1/busco.

**Table 2.** Chromosomal pseudomolecules in the genome assembly of *Tetanocera ferruginea*, idTetFerr1.

INSDC accession	Chromosome	Length (Mb)	GC%
OY282640.1	1	172.08	32.5
OY282641.1	2	166.8	32.5
OY282642.1	3	161.58	32.0
OY282643.1	4	135.73	32.0
OY282644.1	5	133.91	32.0
OY282645.1	X	9.94	32.0
OY282646.1	Y	3.92	33.5
OY282647.1	MT	0.02	28.5

The estimated Quality Value (QV) of the final assembly is 57.2 with *k*-mer completeness of 99.99%, and the assembly has a BUSCO v5.3.2 completeness of 97.7% (single = 96.8%, duplicated = 0.9%), using the diptera_odb10 reference set ( *n* = 3,285).

Metadata for specimens, barcode results, spectra estimates, sequencing runs, contaminants and pre-curation assembly statistics are given at https://links.tol.sanger.ac.uk/species/320963.

## Methods

### Sample acquisition and nucleic acid extraction

A male *Tetanocera ferruginea* (specimen ID Ox002718, ToLID idTetFerr1) was collected from Wytham Woods, Oxfordshire (biological vice-county Berkshire), UK (latitude 51.76, longitude –1.34) on 2022-06-14. The specimen was collected by Liam Crowley (University of Oxford) and Steven Falk (independent researcher) and identified by Steven Falk and preserved on dry ice.

The workflow for high molecular weight (HMW) DNA extraction at the Wellcome Sanger Institute (WSI) includes a sequence of core procedures: sample preparation; sample homogenisation; DNA extraction; HMW DNA fragmentation; and fragmented DNA clean-up. The sample was prepared for DNA extraction at the WSI Tree of Life laboratory: the idTetFerr1 sample was weighed and dissected on dry ice with tissue set aside for Hi-C sequencing ( https://dx.doi.org/10.17504/protocols.io.x54v9prmqg3e/v1). Tissue from the whole organism was disrupted using a Nippi Powermasher fitted with a BioMasher pestle ( https://dx.doi.org/10.17504/protocols.io.5qpvo3r19v4o/v1). DNA was extracted at the WSI Scientific Operations core using the Qiagen MagAttract HMW DNA kit, according to the manufacturer’s instructions.

Protocols developed by the Tree of Life laboratory are publicly available on protocols.io ( https://dx.doi.org/10.17504/protocols.io.8epv5xxy6g1b/v1).

### Sequencing

Pacific Biosciences HiFi circular consensus DNA sequencing libraries were constructed according to the manufacturers’ instructions. DNA sequencing was performed by the Scientific Operations core at the WSI on a Pacific Biosciences SEQUEL II (HiFi) instrument. Hi-C data were also generated from remaining tissue of idTetFerr1 using the Arima2 kit and sequenced on the Illumina NovaSeq 6000 instrument.

### Genome assembly, curation and evaluation

Assembly was carried out with Hifiasm ( Cheng *et al.*, 2021) and haplotypic duplication was identified and removed with purge_dups ( Guan *et al.*, 2020). The assembly was then scaffolded with Hi-C data ( Rao *et al.*, 2014) using YaHS ( Zhou *et al.*, 2023). The assembly was checked for contamination and corrected as described previously ( Howe *et al.*, 2021). Manual curation was performed using HiGlass ( Kerpedjiev *et al.*, 2018) and Pretext ( Harry, 2022). The mitochondrial genome was assembled using MitoHiFi ( Uliano-Silva *et al.*, 2023), which runs MitoFinder ( Allio *et al.*, 2020) or MITOS ( Bernt *et al.*, 2013) and uses these annotations to select the final mitochondrial contig and to ensure the general quality of the sequence.

A Hi-C map for the final assembly was produced using bwa-mem2 ( Vasimuddin *et al.*, 2019) in the Cooler file format ( Abdennur & Mirny, 2020). To assess the assembly metrics, the *k*-mer completeness and QV consensus quality values were calculated in Merqury ( Rhie *et al.*, 2020). This work was done using Nextflow ( Di Tommaso *et al.*, 2017) DSL2 pipelines “sanger-tol/readmapping” ( Surana *et al.*, 2023a) and “sanger-tol/genomenote” ( Surana *et al.*, 2023b). The genome was analysed within the BlobToolKit environment ( Challis *et al.*, 2020) and BUSCO scores ( Manni *et al.*, 2021; Simão *et al.*, 2015) were calculated.

Table 3 contains a list of relevant software tool versions and sources.

**Table 3.** Software tools: versions and sources.

Software tool	Version	Source
BlobToolKit	4.2.1	https://github.com/blobtoolkit/blobtoolkit
BUSCO	5.3.2	https://gitlab.com/ezlab/busco
Hifiasm	0.16.1-r375	https://github.com/chhylp123/hifiasm
HiGlass	1.11.6	https://github.com/higlass/higlass
Merqury	MerquryFK	https://github.com/thegenemyers/MERQURY.FK
MitoHiFi	3	https://github.com/marcelauliano/MitoHiFi
PretextView	0.2	https://github.com/wtsi-hpag/PretextView
purge_dups	1.2.5	https://github.com/dfguan/purge_dups
sanger-tol/genomenote	v1.0	https://github.com/sanger-tol/genomenote
sanger-tol/readmapping	1.1.0	https://github.com/sanger-tol/readmapping/tree/1.1.0
YaHS	1.2a.2	https://github.com/c-zhou/yahs

### Wellcome Sanger Institute – Legal and Governance

The materials that have contributed to this genome note have been supplied by a Darwin Tree of Life Partner. The submission of materials by a Darwin Tree of Life Partner is subject to the **‘Darwin Tree of Life Project Sampling Code of Practice’**, which can be found in full on the Darwin Tree of Life website here. By agreeing with and signing up to the Sampling Code of Practice, the Darwin Tree of Life Partner agrees they will meet the legal and ethical requirements and standards set out within this document in respect of all samples acquired for, and supplied to, the Darwin Tree of Life Project. 

Further, the Wellcome Sanger Institute employs a process whereby due diligence is carried out proportionate to the nature of the materials themselves, and the circumstances under which they have been/are to be collected and provided for use. The purpose of this is to address and mitigate any potential legal and/or ethical implications of receipt and use of the materials as part of the research project, and to ensure that in doing so we align with best practice wherever possible. The overarching areas of consideration are:

•     Ethical review of provenance and sourcing of the material

•     Legality of collection, transfer and use (national and international) 

Each transfer of samples is further undertaken according to a Research Collaboration Agreement or Material Transfer Agreement entered into by the Darwin Tree of Life Partner, Genome Research Limited (operating as the Wellcome Sanger Institute), and in some circumstances other Darwin Tree of Life collaborators.

## Data Availability

European Nucleotide Archive: *Tetanocera ferruginea* (common buff snailkiller). Accession number PRJEB61619; https://identifiers.org/ena.embl/PRJEB61619 ( Wellcome Sanger Institute, 2023). The genome sequence is released openly for reuse. The *Tetanocera ferruginea* genome sequencing initiative is part of the Darwin Tree of Life (DToL) project. All raw sequence data and the assembly have been deposited in INSDC databases. The genome will be annotated using available RNA-Seq data and presented through the Ensembl pipeline at the European Bioinformatics Institute. Raw data and assembly accession identifiers are reported in Table 1.

## Author information

Members of the University of Oxford and Wytham Woods Genome Acquisition Lab are listed here: https://doi.org/10.5281/zenodo.7125292.

Members of the Darwin Tree of Life Barcoding collective are listed here: https://doi.org/10.5281/zenodo.4893703.

Members of the Wellcome Sanger Institute Tree of Life programme are listed here: https://doi.org/10.5281/zenodo.4783585.

Members of Wellcome Sanger Institute Scientific Operations: DNA Pipelines collective are listed here: https://doi.org/10.5281/zenodo.4790455.

Members of the Tree of Life Core Informatics collective are listed here: https://doi.org/10.5281/zenodo.5013541.

Members of the Darwin Tree of Life Consortium are listed here: https://doi.org/10.5281/zenodo.4783558.
