# Supplementary material for: The genome sequence of the common buff snailkiller, Tetanocera ferruginea (Fallén, 1820)
Source: Wellcome Open Res. 2023 Nov 20;8:535. [Version 1] doi: 10.12688/wellcomeopenres.20366.1 (PMC10904942; doi:10.12688/wellcomeopenres.20366.1)
Supplement: Peer review history

# Reviewer report: Reviewer response for version 1

**DOI:** 10.21956/wellcomeopenres.22552.r73553

**Published:** 2024-02-20

**Authors:** Alex Makunin

**Affiliations:** 1 Wellcome Sanger Institute, Hinxton, England, UK

The manuscript by Crowley et al provides a high-quality reference genome for the dipteran species *Tetanocera ferruginea*. 

 My only concern is that's unclear which tissue was used for Hi-C library preparation.

 It is also a good sign that the relative sizes and morphology of the autosomes and sex chromosomes agree well with cytogenetic data in Fig 17 of Boyes et al., 1972.

Are sufficient details of methods and materials provided to allow replication by others?

Yes

Is the rationale for creating the dataset(s) clearly described?

Yes

Are the datasets clearly presented in a useable and accessible format?

Yes

Are the protocols appropriate and is the work technically sound?

Yes

Reviewer Expertise:

chromosome evolution, bioinformatics methods

I confirm that I have read this submission and believe that I have an appropriate level of expertise to confirm that it is of an acceptable scientific standard.

## References

[1] : Further cytotaxonomic studies of Sciomyzidae, with description of a new species, Dichetophora boyesi Steyskal (Diptera: Acalyptratae). *Genetica* .1972;43(3) : 10.1007/BF00156131 334-365 10.1007/BF00156131

# Reviewer report: Reviewer response for version 1

**DOI:** 10.21956/wellcomeopenres.22552.r73557

**Published:** 2024-02-20

**Authors:** Markus Friedrich

**Affiliations:** 1 Wayne State University, Detroit, Michigan, USA

This contribution reports the sequencing and chromosome-scale assembly of the genome sequence of the Tetanocera ferruginea, a dipteran species. Experimental design, documentation, and the genome data produced represent state-of-the-art rigor and quality.

**Minor editorial points:**

**1.** The narrative cites specific work with the authors in and outside parentheses. Examples:

 Sciomyzidae by (Berg, 1953),...

  provided in Foote (1999),...

 The manuscript could be looked over for consistency.

**2.**“More recent population genetics work has attempted to study Tetanocera ferruginea but failed to produce sufficient specimens.”

 Personal observation by the authors?

 If so, state in parentheses.

Are sufficient details of methods and materials provided to allow replication by others?

Yes

Is the rationale for creating the dataset(s) clearly described?

Yes

Are the datasets clearly presented in a useable and accessible format?

Yes

Are the protocols appropriate and is the work technically sound?

Yes

Reviewer Expertise:

Comparative genomics

I confirm that I have read this submission and believe that I have an appropriate level of expertise to confirm that it is of an acceptable scientific standard.

# Reviewer report: Reviewer response for version 1

**DOI:** 10.21956/wellcomeopenres.22552.r73545

**Published:** 2024-02-16

**Authors:** Darren J Obbard

**Affiliations:** 1 The University of Edinburgh, Edinburgh, Scotland, UK

This data note reports the sequencing and assembly of the genome of *Tetanocera ferruginea *as part of the “Darwin Tree of Life” programme. In common with other data notes from this research effort, the reporting is standardised and quite brief. As such, I have very few comments to make.

 The approach is state-of-the-art, the raw data appear to be of a suitably high quality, and the assembly methods are appropriate. The public availability of raw data and genome assembly are appropriate. The resulting genome is likely to be of very high quality, and I have no doubt that it will be of great value to any researchers working on this group of flies, or on the comparative or evolutionary genomics of insects more generally.

 My suggestions for improvement all pertain to the writing of the text, which is sometimes oddly structured and hard to read.

- It would be nice if the article included a better (live, habitus) picture, in addition to the one of the sequenced specimen.
- First line: “… in the dipteran family Sciomyzidae by (Berg, 1953) …” should be formatted as “… in the dipteran family Sciomyzidae by Berg (1953) …”
- It might be nice to mention that this appears to be one of the species cited as an example by Berg (1953),
- At 75 words, the first sentence is unusually long and hard to read.
- Writing Structure: “The biology of *T. ferruginea* is discussed in some detail in … ” – it would be useful to provide a 1-3 sentence summary of this biology here, citing those references.
- Writing Structure:  “This Group is defined as follows: …” I do not feel it is appropriate to quote previous papers in this way. The information should be synthesised / summarised here for the reader.
- In addition, I think the note would benefit from further links to the research literature (see reference below).
- In general, more explicit information on the ecological, temporal, and host range in the would be appreciated.

Are sufficient details of methods and materials provided to allow replication by others?

Yes

Is the rationale for creating the dataset(s) clearly described?

Yes

Are the datasets clearly presented in a useable and accessible format?

Yes

Are the protocols appropriate and is the work technically sound?

Yes

Reviewer Expertise:

Metagenomics, genomics, phylogenetics, and population genetics of invertebrates and their parasites.

I confirm that I have read this submission and believe that I have an appropriate level of expertise to confirm that it is of an acceptable scientific standard.

## References

[1] : Thermal effects on the egg stage of four sciomyzids with reference to phenology and biocontrol potential. *Journal of Applied Entomology* .2007;131(2) : 10.1111/j.1439-0418.2006.01109.x 65-70 10.1111/j.1439-0418.2006.01109.x
